# TOTAL OMENTECTOMY IN GASTRIC CANCER SURGERY: IS IT ALWAYS NECESSARY?

**DOI:** 10.1590/0102-672020180001e1425

**Published:** 2019-02-07

**Authors:** Leandro Cardoso BARCHI, Marcus Fernando Kodama Pertille RAMOS, André Roncon DIAS, Osmar Kenji YAGI, Ulysses RIBEIRO-JÚNIOR, Bruno ZILBERSTEIN, Ivan CECCONELLO

**Affiliations:** 1 Discipline of Digestive Surgery, Department of Gastroenterology, Faculty of Medicine, University of São Paulo, São Paulo, SP, Brazil

**Keywords:** Gastric neoplasm, Recurrenc, Lymph node excisio, Gastrectom, Adenocarcinom, Omentum, Neoplasias gástrica, Recidiv, Excisão de linfonod, Gastrectomi, Adenocarcinom, Omento

## Abstract

**Background:**

Traditionally, total omentectomy is performed along with gastric resection and extended lymphadenectomy in gastric cancer (GC) surgery. However, solid evidences regarding its oncologic benefit is still scarce.

**Aim:**

To evaluate the incidence of metastatic omental lymph nodes (LN) in patients undergoing curative gastrectomy for GC, as well as its risk factors and patients’ outcomes.

**Methods:**

All consecutive patients submitted to D2/modified D2 gastrectomy due to gastric adenocarcinoma from March 2009 to April 2016 were retrospectively reviewed from a prospective collected database.

**Results:**

Of 284 patients included, five (1.8%) patients had metastatic omental LN (one: pT3N3bM0; two: pT4aN3bM0; one: pT4aN2M0 and one pT4bN3bM0). Four of them deceased and one was under palliative chemotherapy due relapse. LN metastases in the greater omentum significantly correlated with tumor’s size (p=0.018), N stage (p<0.001), clinical stage (p=0.022), venous invasion growth (p=0.003), recurrence (p=0.006), site of recurrence (peritoneum: p=0.008; liver: p=0.023; ovary: p=0.035) and death (p=0.008).

**Conclusion:**

The incidence of metastatic omental LN of patients undergoing radical gastrectomy due to GC is extremely low. Total omentectomy may be avoided in tumors smaller than 5.25 cm and T1/T2 tumors. However, the presence of lymph node metastases in the greater omentum is associated with recurrence in the peritoneum, liver, ovary and death.

## RESUMO

**Racional:**

Tradicionalmente a omentectomia total é realizada juntamente com a ressecção gástrica associada à linfadenectomia na cirurgia do câncer gástrico. No entanto, evidências sólidas em relação ao seu benefício oncológico são escassas .

**Objetivo**: Avaliar a incidência de metástases em linfonodos do omento maior em pacientes submetidos à gastrectomia potencialmente curativa por câncer gástrico, assim como, avaliar os fatores de risco para a ocorrência e a evolução dos pacientes.

**Métodos:**

Pacientes consecutivos submetidos à gastrectomia D2/D2 modificada devido ao adenocarcinoma gástrico foram analisados retrospectivamente a partir de um banco de dados.

**Resultados:**

Dos 284 pacientes, cinco (1,8%) tinham linfonodos metastáticos no omento maior (um pT3N3bM0; dois pT4aN3bM0; um pT4aN2M0 e um pT4bN3bM0). Quatro faleceram e um estava em tratamento paliativo com quimioterapia devido à recidiva da doença. Os linfonodos metastáticos no omento maior tiveram correlação significativa com o tamanho do tumor (p=0,018), estádio N (p<0,001), estádio clínico (p=0,022), invasão venosa (p=0,003), recorrência (p=0,006), local de recorrência (peritônio p=0,008; fígado p=0,023; ovário p=0,035) e óbito (p=0,008).

**Conclusão:**

A incidência de linfonodos metastático no omento maior de pacientes submetidos à gastrectomia radical por câncer gástrico é baixa. A omentectomia total pode ser evitada em tumores menores que 5,25 cm e estádios T1/T2. Entretanto, a presença de metástases linfonodais no omento maior está associada à recidiva no peritônio, fígado, ovário e óbito.

## INTRODUCTION

The prognostic relevance of the multimodal treatment in gastric cancer (GC) has been well stablished^30^. However, surgery remains the only possibility of cure, especially when is associated with early diagnosis^18^ ^,^ ^32^. It is also known that the lymph node (LN) involvement is the main prognostic factor after potential curative resection (R0)^28^. Thus, systematic removal of LN is considered crucial in GC surgery and the number of harvested LN as a direct measure of the quality of surgery^3^ ^,38^. 

The greater omentum (including the omental sac) is removed in block among with LN in this type of operation. It is believed that total omentectomy (TO) is essential to ensure the elimination of cancer cells during advanced GC surgery^11^. However, the greater omentum plays an important role in the peritoneal primary defense. Therein are the milky spots (mesenchymal cells covered by a mesothelium layer containing macrophages (70%), B lymphocytes (10%), T lymphocytes (10%) and mast cells. The omentum reduces intestinal adhesions and prevents free peritonitis. Patients who undergo to TO are more vulnerable to peritoneal infections, which are often associated with worse clinical outcomes^4^ ^,^ ^26^. Further, it has been reported that long-term overall survival (OS) does not differ between patients undergoing total or partial omentectomy (PO), whereas the incidence of complications is higher in the TO patients^9^ ^,^ ^36^. 

There is no consensus regarding the oncologic value of omentectomy in GC surgery between the European, American and Japanese guidelines. The European guideline provides no guidance on this subject^34^, whereas the American guidelines recommend resection of the greater and lesser omentum^1^. Alternatively, the Japanese Gastric Cancer Association (JGCA) guidelines recommend the preservation of the omentum 3 cm distal to gastroepiploic vessels in patients with T1/T2 stage tumors and TO in T3/T4 tumors^19^. Its pivotal role is not discussed when there is a suspicion or evident metastatic invasion of the greater omentum. However, the unnecessary impact of TO might be substantial.

In this context, over the past few years, laparoscopic gastrectomy has become an alternative procedure for early GC patient^19^. Studies are underway to confirm its efficacy in more advanced forms^16^ ^,^ ^17^. In open surgery, the dissection of the greater omentum through the avascular plane of the transverse colon can lead to TO quickly and satisfactorily. However, the complete resection of the greater omentum during laparoscopic gastrectomy is associated with longer operative time and higher risks of adjacent organs injury, without interfering in the recurrence or in the disease specific survival^21^. 

There is a possibility that the LN in the greater omentum may be spared of metastatic involvement in GC patients. Therefore, this part of the procedure could be omitted, mainly in minimally invasive surgery, reducing surgical time and avoiding complications. 

This study aimed to evaluate the incidence of metastatic LN in the greater omentum of patients who underwent radical gastrectomy for GC, as well as its risk factors and patients’ outcomes.

## METHODS

The study was approved by the hospital ethics committee (NP898/2016) and registered at “Plataforma Brasil” (CAAE: 56307516.9.0000.0068) that collects all research projects that involve human beings in country. 

### Patients

We retrospectively reviewed all patients submitted to R0 gastrectomy due to gastric adenocarcinoma from March 2009 to April 2016 from a prospective collected database. Patients with gastric stump neoplasia, histological type different from adenocarcinoma, macroscopic metastatic involvement of the greater omentum (carcinomatosis), distant metastasis and emergency surgeries were excluded from the analysis. 

Patients were staged in the preoperatively through abdominal/pelvis computed tomography, endoscopy and laboratory tests. Extension of gastric resection (total x subtotal) was based on the location of the tumor to obtain free proximal margin^37^. TNM staging was performed according to the TNM 7^th^ edition^8^. 

All cases were operated in a high-volume center by surgeons with extensive experience in the surgical management of GC. The surgical technique, extension of resection and dissection of LN chains followed the recommendations of the JGCA guidelines^19^. Total omentectomy and D2 lymphadenectomy was performed in all patients. Digestive tract was reconstructed through Roux-en-Y anastomosis. The survival status was assessed during follow-up. Patients without medical consultations for over one year were considered as the loss of follow-up. The type of recurrence was classified as peritoneal, local (lymph node or anastomosis) and distant (liver, ovary, lung, bone and others).

### Pathological analysis

**FIGURE 1:**
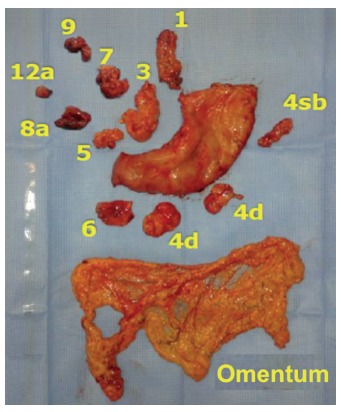
Surgical specimen of subtotal gastrectomy with the greater omentum and lymph node chains dissected and sent in separate for histopathological analysis

Routinely, at the end of each operation, the surgical specimen was prepared by a member of the surgical team. The omentum was divided from the specimen distally to the gastroepiploic vessels (LN station number 4, Figure 1). The LN stations and the omentum were sent in separate flasks for histopathological analysis. The materials were fixed in 10% formaldehyde for 24-48 h, and then analyzed. The tumor was described in order to characterize its size, location (antrum, body, proximal or diffuse), the Borrmann classification and the resection margins. All material was meticulously evaluated macroscopically, particularly to seek for implants. Through the visualization, palpation and section, the LN stations and the greater omentum were dissected to search for LN. When they were found, were described in terms of number, size and external macroscopic appearance. The LN larger than 0.6 cm were sliced and shown alone in a paraffin block. The other LN were represented together in a paraffin block. If suspected implant area was visualized, this area was described and represented. If no change was seen, about 3-5 cuts were performed. 

The slides were stained by the H&E method and evaluated by the pathologist in a conventional optical microscope. 

### Statistical analysis

The numerical variables were described by mean and standard deviation (SD) or median and quartiles, minimum and maximum values. The categorical variables were described by absolute and relative frequencies. The association between the LN involvement in the greater omentum with categorical variables was investigated by Fisher’s exact test or chi-square test and numerical variables by Wilcoxon-Mann-Whitney tests. The ROC (Receiver Operating Characteristic) curve was constructed to determine the cut-off of tumor’s size. The analysis was performed by SPSS^®^ software version 18, adopting the significance level of 5%. 

## RESULTS

Of the total of 346 patients, 62 (17.9%) were excluded, who did not meet the inclusion/exclusion criteria of the study and were: 26 (41.9%) because they underwent degastrectomy; 12 (19.7%) had no adenocarcinoma (histopathological analysis revealed GIST and neuroendocrine tumor); eight (13.1%) had less than 15 LN harvested in the operation; seven (11.4%) had no tumor found in anatomopathological analysis (post-margins compromised in endoscopic resections); five (8.2%) had direct invasion of the omentum, three (4.9%) had distant metastasis (M1) and one (1.6%) had a synchronous tumor (colon neoplasia). After the exclusions, the study sample consisted of 284 patients.

**TABLE 1:** Baseline characteristics and perioperative results (n=284)

Gender	n (%)
Male	159 (55.9)
Female	125 (44.1)
BMI kg/m^2^ (min-max)	24.6 (14 - 46.5)
Resection Subtotal	182 (64.1)
Total	102 (35.9)
Approach	
Conventional	253 (89.1)
Laparoscopic	16 (5.6)
Robotic	7 (2.5)
Hybrid	8 (2.8)
Site of tumor	
Diffuse	6 (2.1)
Antrum	185 (65.2)
Body	55 (19.3)
Proximal	38 (13.3)
Borrmann classification ^*^ I	28 (9.8)
II	55 (19.3)
III	135 (47.5)
IV	56 (19.7)
Neoadjuvant chemotherapy Yes	40 (14.1)
No	244 (85.9)
Multivisceral resection #	
Yes	36 (12.6)
No	248 (87.4)
Mortality	
30-day	9 (3.1)
90-day	16 (5.6)

(%); *10 patients not classified; #: details are shown in Table 2

**TABLE 2:** Surgery with multivisceral resection in the sample (n=284)

Mesocolon - anterior sheet	n (%)
No	90 (31.6)
Incomplete	29 (10.2)
Yes	165 (58.1)
Pancreatic capsule	
No	96 (33.8)
Incomplete	37 (13)
Yes	151 (53.1)
Oncologic splenectomy	
No	270 (95.1)
Yes	14 (4.9)
Tactical splenectomy	
No	282 (99.2)
Yes	2 (0.8)
Hepatectomy	
No	281 (98.9)
Yes	3 (1.1%)
Colectomy	
No	278 (97.8)
Yes	6 (2.2)
Pancreatectomy	
No	274 (96.4)
Yes	10 (3.6)
Resection of the diaphragm	
No	283 (99.6)
Yes	1 (0.4)

**FIGURE 2:**
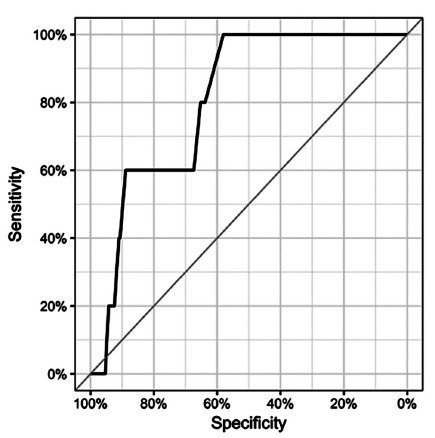
The area under the ROC curve (0.8072 - (IC95%:0.6645 - 0.9498) shows that patients with metastatic LN in the omentum have larger tumors

There was a male preponderance of 159 (55.9%) patients, with a mean age of 61.8 years (±11.9; 25-86). The mean body mass index (BMI) was 24.6 kg/m^2^ (±4.7; 14-46.5). Subtotal gastrectomy was performed in 182 (64.1%). The tumor was located at the antrum in 185 (65.2%). Forty (14.1%) patients received neoadjuvant chemotherapy. Open surgery was performed in 253 (89.1%). Thirty-day mortality was 3.1% (nine patients). The median follow-up period for all patients was 27.6 months (1-89.5). The median follow-up time of the disease-free patients was 34.3 months (1-89.5). The baseline characteristics of patients and the perioperative results are shown in Table 1. The pathological analysis is summarized in Table 3. Peritoneal washing was negative in all patients. The average number of LN resected was 41.2 (±17; 15-114). The average number of positive LN was 4.69 (±8.12; 0-53). The Lymph Node Ratio (LNR) was 0.113 (±0.447; 0-0.96). The intestinal histological type of Lauren occurred in 146 (51.4%) patients. Poorly differentiated tumors were also 146 (51.4%). Sixty-six (23.2%) had at least one LN in the greater omentum. Metastatic LN were found in five (1.8%) patients (one: pT3N3bM0; two: pT4aN3bM0; one: pT4aN2M0 and one pT4bN3bM0). The mean size of tumors of patients without metastatic omental LN was 4.8 cm (±2.96; 0.5-14.5), while the mean size of tumors of patients with metastatic LN was 8.06 cm (±2.75; 2.75-9, p=0.018). The cut-off point was 5.25 cm (area under the curve: 0.8072; IC95%: 0.6645-0.9498, Figure 2). Metastatic LN in the greater omentum was significantly correlated with N stage (p<0.001), clinical stage (p=0.022) and venous invasion growth (p=0.003). 

**TABLE 3:** Pathological analysis according to lymph node involvement in the greater omentum

		Metastatic omental LN		
	Total (n=284)	No (n=279)	Yes (n=5)	
pT stage				
T1/T2	116	116 (41.6)	0 (0)	p_1_=0.081
T3/T4	168	163 (58.4)	5 (100)	
pN stage				
N0 / N1	162	162 (57.8)	0 (0)	p_1_<0.001
N2	60	59 (21.1)	1 (20)	
N3a	36	36 (12.9)	0 (0)	
N3b	27	23 (8.2)	4 (80)	
Clinical stage				
I / II / IIIA	205	205 (73.5)	0 (0)	p_1_=0.022
IIIB	41	39 (13.9)	2 (40)	
IIIC	38	35 (12.5)	3 (60)	
Tumor size (cm) ^&^	4.3	4.8	8.06	p_1_= 0.018
Lauren classification				
Intestinal	146	145 (52)	1 (20)	
Diffuse	108	105 (37.6)	3 (60)	p_1_=0.290
Mixed	30	29 (10.4)	1 (20)	
Differentiation grade				
Well differentiated	22	21 (7.5)	1 (20)	
Mod. differentiated	116	116 (41.6)	0 (0)	p_1_=0.098
Poorly differentiated	146	142 (50.9)	4 (80)	
Lymphatic invasion growth *				
Yes	143	138 (49.5)	5 (100)	p_1_=0.060
No	140	140 (50.5)	0 (0)	
Venous invasion growth *				
Yes	48	44 (15.8)	4 (80)	p_1_=0.003
No	235	234 (82.2)	1 (20)	
Perineural invasion growth *				
Yes	140	136 (48.7)	4 (80)	p_1_=0.211
No	143	142 (51.3)	1 (20)	
Intestinal metaplasia				
Yes	133	130 (46.6)	3 (60)	p_3_= 0.552
No	151	149 (53.4)	2 (40)	
Tumor site				
Antrum	28	182 (65.2)	3 (60)	
Body	55	55 (19.7)	0 (0)	p_1_=0.279
Proximal	135	36 (12.9)	2 (40)	
Diffuse	56	6 (2.1)	0 (0)	
Neoadjuvant therapy				
Yes	40	39 (14)	1 (20)	
No	244	240 (86)	4 (80)	
Gastrectomy				
Subtotal	182	179 (64.1)	3 (60)	p_1_>0.999
Total	102	100 (35.9)	2 (40)	
Omental size (cm^3^) ^&^	-	720 (11.5-6,075)	1,087 (595-3,81)	p_2_=0.331
Harvested LN ^&^	41.2 (15-114)	41.1 (15-114)	47.2 (31-73)	p_2_=0.332
Positive harvested LN ^&^	4.7 (0-53)	4.4 (0-53)	20.8 (4-31)	p_2_=0.001
Patients with omental LN ^&^	66 (23)	61 (22)	5 (100)	
No. omental LN ^&^	0 (1-6)	0 (1-6)	0 (1-3)	
No. positive omental LN	5	0	5 (100)	

(%); p_1_=Fisher’s exact test; p_2_=Mann-Whitney’s test; p_3_=Qui-square test; *=one patient undetermined; &=values are mean(range)

Cutt-off=5.25 cm; specificity=65.2%; sensitivity= 80%; negative predictive value=99.4%; positive predictive value=3.96%; accuracy=65.4%

During the follow-up period 163 (57.4%) were free of the disease. The cancer relapse was found in 65 (22.8%) patients and the most frequent site was in the peritoneum (46.2%). Four patients with metastatic omental LN died and the other one was under palliative chemotherapy due to the relapse on the liver and pleura. We found association between metastatic LN in the omentum with recurrence (p=0.006), site of recurrence (peritoneum: p=0.008; liver: p=0.023; ovary: p=0.035) and death (p=0.008, Table 4).

Patients who did not relapse prior to loss of follow-up or in whom this data was unavailable were removed from the sample. For this analysis, 228 patients remained. 

**TABLE 4:** Correlation between the presence of metastatic lymph nodes in the omentum and patient status

	Metastatic omental LN		
Patient status	No (n=224)	Yes (n=4)^*^	
Recurrence			
No	163 (72.8)	0 (0)	p=0.006
Yes	59 (27.2)	4 (100)	
Peritoneal			
No	197 (87.9)	1 (25)	p=0.008
Yes	27 (12.1)	3 (75)	
Local			
No	196 (87.5)	4 (100)	p>0.999
Yes	28 (12.5)	0 (0)	
Anastomosis			
No	222 (99.1)	4 (100)	p>0.999
Yes	2 (0.9)	0 (0)	
Lymph node			
No	199 (88.8)	4 (100)	p>0.999
Yes	25 (11.2)	0 (0)	
Distant			
No	205 (91.5)	1 (25)	p=0.003
Yes	19 (8.5)	3 (75)	
Liver			
No	211 (94.2)	2 (50)	p=0.023
Yes	13 (5.8)	2 (50)	
Lung			
No	222 (99.1)	3 (75)	p=0.052
Yes	2 (0.9)	1 (25)	
Ovary			
No	223 (99.5)	3 (75)	p=0.035
Yes	1 (0.5)	1 (25)	
Bone			
No	222 (99.1)	4 (100)	p>0.999
Yes	2 (0.9)	0 (0)	
Others ^#^			
No	221 (98.7)	3 (75)	p=0.069
Yes	3 (1.3)	1 (25)	
Outcome			
Free of disease	163 (72.7)	0(0)	
Relapse under treatment	14 (6.3)	1 (25)	
Death	78 (34.8)	4 (75)	p=0.008
Loss of follow-up	24 (10.7)	0 (0)	

p=Fisher’s exact test; (%); #=brain, pleura; ^*^=the cause of death is unknown in one patient with positive lymph node in the omentum and therefore could not be attributed to disease relapse

## DISCUSSION

Despite the great advances of the multimodal therapy that have led to greater OS, free-margin gastrectomy associated with adequate lymphadenectomy remain crucial components in GC surgery with curative intent. Once questioned, extended lymphadenectomy (D2) provides better local control of the disease, allows accurate staging and avoids the stage migration phenomenon. In addition, surgery leads to better overall long-term survival.^6^ ^,^ ^7^ ^,^ ^31^

Traditionally, TO is performed as a part of subtotal/total gastrectomy with lymphadenectomy. The complete removal of the greater omentum has been considered essential to ensure the elimination of micrometastasis^23^. However, there is no consensus regarding the real benefit of TO on survival improvement and relapse decrement. 

Several experimental studies have reported that cancer cells sown in the peritoneal cavity preferentially grow in the omentum, specifically at the milky spots^12^ ^,^ ^27^. Besides, many researchers insist that the extra nodal expansion occurs in some metastatic LN, which means that cancer cells spread from the LN capsule to adjacent adipose tissue^24^. 

Conversely, the greater omentum contains zones with high concentrations of immune system cells that can contribute to remove foreign bodies and bacteria^33^. Yet, it reduces the possibility of intestinal adhesions, not only by creating a mechanical barrier between the bowel and the abdominal wall, but also due to the production of fibrinolytic factors by the mesenchymal cells^5^. Other possible advantages of the omental preservation are the decrease in operative time (mainly in minimally invasive surgery), the blood loss and the reduction of complications such as abdominal abscess, ascites, anastomotic leakage, ileus, wound infection and iatrogenic lesions of the mesocolon and colon^10^ ^,^ ^14^ ^,^ ^22^. Moreover, several studies have demonstrated no difference in OS nor disease free survival (DFS) between total and partial omentectomy in GC surgery^10^ ^,^ ^13^ ^,^ ^14^ ^,^ ^21^ ^,^ ^22^. Kim *et al*.^21^ compared TO with PO in 146 patients operated by laparoscopy for advanced GC. Propensity score match analysis (T and N parameters) showed no difference between the groups regarding DFS (TO versus PO: 83.3% vs. 90.5%, p=0.442). 

Although that there are some studies that compared short/long-term outcomes, complications, relapse and survival between TO and PO in GC surgery, specific studies regarding the incidence of metastatic omental LN are lacking. Haverkamp *et al.* ^*15*^ prospectively evaluated the presence of omental LN and tumor deposits in 50 patients undergoing gastrectomy for GC. One (2%) had metastatic omental LN (stage IB) and four (8%) omental tumor deposits (stages IB, IIA, IIB and IIIA). Patients with tumor deposits had significantly reduced 1-year DFS compared to patients without tumor deposits (0 vs. 58.7%, p=0.003). However, no significant difference in 1-year OS of was found (25.0 vs. 67.4%, respectively, p=0.079). The authors did not find any predictive factors for omental metastasis^31^. 

On the other hand, another prospective trial named OMEGA analyzed the presence of omental metastasis in 100 patients. Metastasis were detected in five (5%) patients (two with metastatic LN and three with tumor cell deposits). All of them were at least stage pT3 with macroscopically non-radical resection (p<0.001). Yet, omental metastasis was also significantly correlated with linitis plastica or location in the proximal third of the stomach (p=0.002), tumor diameter of 5 cm or larger, stage III-IV disease (p=0.010) and (y)pM1 category (p*<*0.001)^20^. 

The present study corroborates the OMEGA trial results. It was found a significant correlation with tumors larger than 5.25 cm. Still, as well as the Japanese guidelines, all patients with omental LN metastasis were categorized as stage pT3/T4, which allows us to state that TO may be omitted in stage T1/T2 tumors. In fact, patients with positive omental LN had very advanced disease and there was a significant correlation with N stage (p<0.001) and clinical stage (p=0.022). Nevertheless, the vast majority of pT3/T4 tumors were free of metastatic omental LN (pT3: 1 positive/93 negative; pT4: 4 positive/75 negative), suggesting that TO may be also avoided in many T3/T4 tumors. The heart of the matter is how to identify these patients preoperatively, as only tumor’s size was the only risk factor associated with omental disease. Other significant parameters such as venous invasion growth (p=0.003) could only be assessed postoperatively. The rarity of the incidence also suggests that other conditions may be involved with omental LN disease. 

**FIGURE 3:**
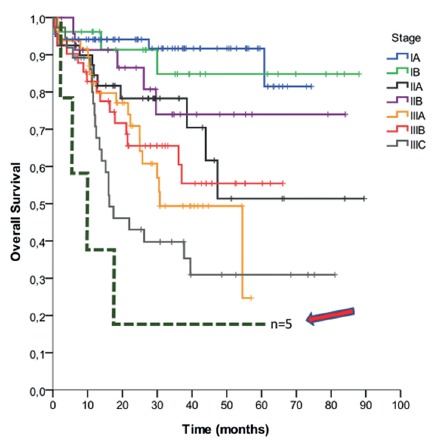
Kaplan-Meier curve of survival: each line represents the OS of patients within a single clinical stage (TNM 7^th^ edition). The arrow indicates patients with omental metastatic LN

Another interesting aspect is the fact that omental LN metastasis was associated with recurrence (p=0.006), site of recurrence (peritoneum: p=0.008; liver: p=0.023; ovary: p=0.035) and death (p=0.008). Figure 3 represents OS curve of patients according to clinical stage and patients with omental LN metastasis. It may be alleged that, when there is omental LN involvement, systemic disease is in course, with very dismal prognosis. The purpose of performing TO is to remove micro metastasis and therefore to avoid mostly peritoneal recurrence. As in all patients in this cohort TO was performed, it can be speculated that removing the entire omentum during radical gastrectomy does not prevent relapse and, above all, death. 

This study has some limitations. Firstly, five patients (three: stage pT4aN3aM1; one: pT4bN3M0 and one: pT3N2M0) with tumor deposits in the greater omentum were not included. There was not enough information whether metastatic omental involvement was diagnosed during surgery or through pathological analysis. Obviously, apart from identifying metastatic LN, to identify tumor deposits is essential to achieve R0 resection. In any case, the staging of these patients confirms that the omental disease is associated with advanced disease. Secondly, the oncotic cytology of peritoneal lavage was negative in all patients, similar to OMEGA trial. Some studies indicate up to 10% incidence of positive cytology in patients without peritoneal metastases^25^ ^,^ ^29^. Peritoneal cytology could be obtained in diagnostic laparoscopy and, certainly, would influence the decision to proceed with TO and possibly offer more aggressive treatments such as hyperthermia intraperitoneal chemotherapy, which is now under evaluation^2^ ^,^ ^35^. 

## CONCLUSION

The incidence of metastatic omental LN of patients undergoing radical gastrectomy due to GC is extremely low. Total omentectomy may be avoided in tumors smaller than 5.25 cm and T1/T2 tumors. However, lymph node metastasis in the greater omentum is associated with recurrence in the peritoneum, liver, ovary and death. 
